# Diabetes mellitus hospitalization and mortality rate according to a national database in Brazil: a longitudinal study

**DOI:** 10.1186/s12889-021-10438-z

**Published:** 2021-02-25

**Authors:** Rêncio Bento Florêncio, Luiza Gabriela de Araújo Fonseca, Vivian Fernanda Dantas da Silva, Íllia Nadinne Dantas Florentino Lima, Lucien Peroni Gualdi

**Affiliations:** 1 Mestrando do Programa de Ciências da Reabilitação da Faculdade de Ciências da Saúde do Trairí/Universidade Federal do Rio Grande do Norte – FACISA/UFRN, Santa Cruz, RN Brazil; 2 Graduanda do Curso de Fisioterapia da Faculdade de Ciências da Saúde do Trairí/Universidade Federal do Rio Grande do Norte – FACISA/UFRN, Santa Cruz, RN Brazil; 3 Docente da Faculdade de Ciências da Saúde do Trairí/Universidade Federal do Rio Grande do Norte – FACISA/UFRN, Santa Cruz, RN Brazil

**Keywords:** Health services research, Diabetes mellitus, Hospitalization, Hospital costs

## Abstract

**Background:**

Diabetes mellitus (DM) is an important public health problem worldwide. In addition to the impairment in functionality, the large number of complications which lead to hospitalizations results in high treatment costs. The aim of this study was to analyze the incidence of hospitalizations, mortality rate and hospital costs, as well as to observe the temporal trend of hospitalizations and length of hospital stay due to DM between 2008 and 2019 in Brazil.

**Methods:**

This is a longitudinal descriptive study in which all data regarding hospital admissions registered in the Brazilian system of Hospital Information of *“Sistema Único de Saúde”* (*SIH*/*SUS*; http://datasus.saude.gov.br) due to DM (ICD-10) were included. Comparisons among the groups were performed by an unpaired Student’s t-test, two-way ANOVA with a Tukey post hoc test (*p* < 0.05).

**Results:**

An increased hospitalization of 1.83% due to DM was observed between 2008 and 2019 in Brazil. The Southeastern region had the highest incidence (34.6%) and mortality rate when compared to the other regions (*p* < 0.05). We also found that females were more likely to be hospitalized in comparison to males, without a statistically significant difference. Finally, a progressive increase of hospitalizations and mortality rate were observed according to age groups, as well as increased spending due to DM hospitalizations over the years.

**Conclusion:**

Hospitalizations due to DM in Brazil showed an expressive increase over the last 12 years, and there is a need for primary healthcare interventions to help reduce this situation.

## Introduction

Diabetes mellitus (DM) currently appears as an important cause of morbidity and mortality worldwide. It is a public health problem owing to the number of affected individuals and the decrease in functionality, as well as high costs involved in its control and treatment [1, 2]. An estimated 13 million people were diagnosed with DM in Brazil, being ranked the fourth most prevalent country in the world. Moreover, it is projected to reach 23 million individuals by the year 2040, while the global prevalence may reach 471 million subjects by 2035, representing approximately 10% of total healthcare spending [3, 4].

The increase in DM prevalence seems to be linked to the world’s aging population. Moreover, reductions in infectious diseases and a relative increase in chronic non-communicable diseases such as DM have been observed in the last decades [5]. In addition to genetic inheritance, other extrinsic factors such as lifestyle changes, obesity, dyslipidemia, insulin resistance, systematic arterial hypertension (SAH), and sedentarism, among others, are closely related to DM [6].

According to the World Health Organization (WHO), the number of DM diagnoses increased by approximately 62% between 2006 and 2016 in Brazil [2]. Moreover, this increase may still be underreported, as many individuals may present the disease without evident symptoms. Based on this scenario, it is essential to know the impacts of exacerbations in hospital admissions as the costs associated with DM reached USD $22 billion in Brazil by 2015, and may reach USD $29 billion by 2040 [3].

Due to the large number of complications which lead to hospitalization, it is possible to predict the great burden that DM may cause on healthcare systems, especially in developing countries such as Brazil. The country already presents an overloaded public health system called the *“Sistema Único de Saúde (SUS)”,* due to other non-communicable chronic diseases. Thus, it is important to investigate hospital admissions and the mortality rate due to DM in the Brazilian territory in recent years, aiming to generate knowledge to increase prevention and to reduce healthcare costs.

The database from the IT Department of the “*Sistema Único de Saúde”* (*DATASUS*) is a free and reliable platform which provides hospital admission information through the hospital information system of the Unified Health System (*SIH*/*SUS*) such as number, costs and mortality. Therefore, the aim of this study was to analyze the hospitalization incidence and mortality rate, as well as to observe the temporal trend of hospitalizations, length of hospital stay and costs due to DM.

## Methods

### Study design

This is a longitudinal descriptive study which utilized data on hospital admissions registered in the Brazilian Hospital Information system of the *“Sistema Único de Saúde”* (*SIH*/*SUS*) due to DM (International Classification of Diseases, ICD-10) [7]. Data from both private or public services from 2008 and 2019 were accessed. All hospitalizations registered with Department of Informatics of the Unified Health System (*DATASUS*) in the period from 2008 to 2019 were included in the study. The diagnosis for DM was made by a medical team in each hospital center distributed in Brazil according to clinical criteria established by the Brazilian Diabetes Society (2015) [8].

### Ethical aspects

Ethics approval is not required in accordance with the Brazilian National Health Council (Resolution No.510 from April 07th, 2016), which regulates the National Research Ethics Committee (*CONEP*) [9]; patients’ confidentiality was preserved. All data are public with free access and may be accessed at *DATASUS* (http://datasus.saude.gov.br/).

### Data extraction

Data were provided by the Health Surveillance Bureau of the Brazilian Ministry of Health through its open access webpage available from *DATASUS* [10]. The following variables were collected: number of hospital admissions and mortality rate classified by the ICD-10. Absolute values and the frequency of hospital admissions were grouped according to sex, age and living region, as well as the number of deaths recorded. All data were provided by the hospital information system through an online platform (link: http://datasus.saude.gov.br/), and were collected in April, 2020.

### Data analysis

Extracted data was stored in a Microsoft Excel version 2016 program spreadsheet. Statistical analysis was performed by the GraphPad Prism version 6.0 software program. Data normality was assessed by the Kolmogorov-Smirnov test. Comparisons among the sex groups were performed by an unpaired Student’s t-test, and two-way ANOVA with a Tukey post hoc test. A *p*-value < 0.05 was considered significant.

## Results

There were 1,560,060 hospitalizations due to DM complications between 2008 and 2019 in Brazil. This number represents 1.50% of the total admissions in the *SIH*/*SUS* system during this period. We observed an increase of 1.83% in absolute number of hospitalizations across the country between 2008 and 2019. Another important finding is that 94.28% (1,470,863) of hospital admissions were considered to be of an urgent nature.

### Hospital admissions according to living region, sex and age group

When we grouped hospital admissions according to Brazilian regions, it was found that the highest hospitalization incidence was observed in the Southeast region (539,078), followed by the Northeast (495,412), South (262,059), North (141,004) and Midwest (122, 507) (*p* < 0.0001), as shown in Table 1.

**Table 1:** Hospitalization incidence, sex and average hospital stay by region

Regions	Hospitalization		Sex (%)		Length of stay	
	Frequency (%)	***p***-value	Male	Female	Mean ± SD	***p*** value
North^a^	141,004 (9.0)	< 0.05 b c d	65,350 (4.2)	75,654 (4.8)	6.3 ± 0.3	< 0.05 c d e
Northeast^b^	495,412 (31.8)	< 0.05 a c d e	213,967 (13.7)	281,445 (18)	6.1 ± 0.4	< 0.05 c d e
Southeast^c^	539,078 (34.6)	< 0.05 a b d e	262,021 (16.8)	277,057 (17.8)	6.7 ± 0.1	< 0.05 a b d e
South^d^	262,059 (16.8)	< 0.05 a b c e	112,730 (7.2)	149,329 (9.6)	5.3 ± 0.1	< 0.05 a b c
Midwest^e^	122,507 (7.9)	< 0.05 b c d	55,819 (3.6)	66,688 (4.3)	5.6 ± 0.5	< 0.05 a b c
Total	1,560,060 (100)		709,887 (45.5)	850,173 (54.5)	6.2 ± 0.2	

Values are expressed in absolute numbers, percentage (%) and mean ± standard deviation. ^a^North; ^b^Northeast; ^c^Southeast; ^d^South and ^e^Midwest; expressing statistically significant differences among regions and length of stay by two-way ANOVA with post hoc Tukey (*p* < 0.05). Source: Ministry of Health - *SUS* Hospital Information System (*SIH*/*SUS*)

**Fig. 1:**
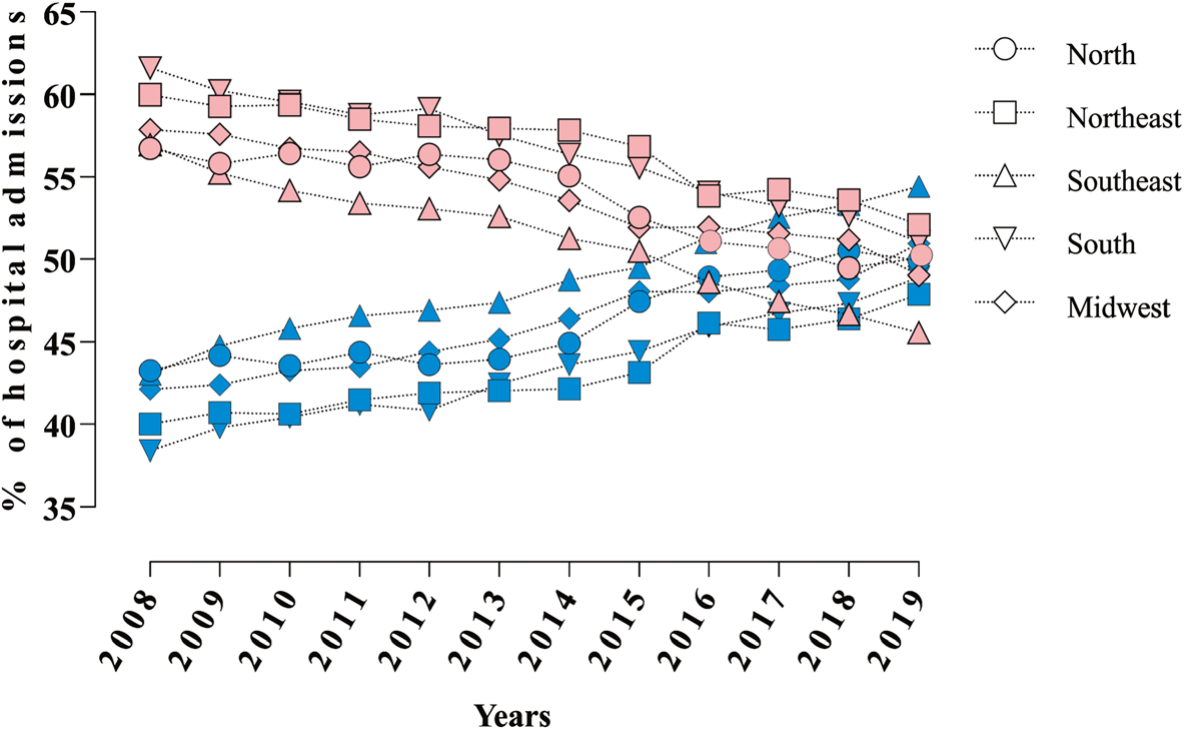
Hospital admissions in males and females in all regions. Values are in percentage. Red symbols: females; Blue symbols: males. Source: Ministry of Health - *SUS* Hospital Information System (*SIH*/*SUS*)

When we analyzed the incidence according to sex, it was found that 54.5% (*n* = 850,173) of hospital admissions occurred in females and 45.5% (*n* = 709,887) in males (*p* < 0.0001). This difference between sexes decreased over the analyzed period in the longitudinal analysis. Moreover, the hospitalization numbers became similar between females and males at the end of the period (i.e. women showed a tendency to reduce, while men increased in the number of hospitalizations over the years), as shown in Fig. 1. A reduction of 14.7% (*n* = 10,710) in hospital admission was observed in females when we compared 2008 and 2019 (*p* < 0.05). On the other hand, males showed an increase of 25.4% (*n* = 12,973) in the comparison for the same period (*p* < 0.05).

In analyzing the number of hospitalizations according to age group, we found a progressive increase according to higher age. It was observed that individuals between 60 and 69 years represented 25.46% (*n* = 397,121) of total hospitalizations, followed by 50–59 years (21.09%), 70–79 years (20.64%), 40–49 years (11.58%), over 80 years (11.05%), 30–39 years (6.13%) and 20–29 years (4.05%). Significant differences between the age groups are shown in Table 2.

**Table 2:** Hospitalization incidence according to age group

Range age	Hospitalization		Length of stay	
	Frequency (%)	***p***-value	Mean ± SD	***p***-value
20–29^a^	63,232 (4.1)	< 0.0001 ^b c d e f g^	5.8 ± 0.1	< 0.05 ^b c d e f g^
30–39^b^	95,568 (6.1)	< 0.0001 ^a c d e f g^	6.0 ± 0.2	< 0.05 ^a c d e^
40–49^c^	180,601 (11.6)	< 0.0001 ^a b d e f^	6.2 ± 0.2	< 0.05 ^a b d f g^
50–59^d^	329,103 (21.1)	< 0.0001 ^a b c e g^	6.4 ± 0.3	< 0.05 ^a b c f g^
60–69^e^	397,121 (25.5)	< 0.0001 ^a b c d f g^	6.3 ± 0.3	< 0.05 ^a b f g^
70–79^f^	322,043 (20.6)	< 0.0001 ^a b c e g^	6.0 ± 0.3	< 0.05 ^a c d e^
80- > 80^g^	172,392 (11.1)	< 0.0001 ^a b d e f^	5.9 ± 0.1	< 0.05 ^a c d e^
Total	1,560,060 (100)		6.2 ± 0.2	

Values are expressed in absolute numbers, percentage (%) and mean ± standard deviation. ^a^20–29 years; ^b^30–39 years; ^c^40–49 years; ^d^ 50–59 year; ^e^60–69 years; ^f^70–79 years and ^g^80- > 80 years; expressing statistically significant differences among regions and length of stay by two-way ANOVA with pos hoc Tukey (*p* < 0.05). Source: Ministry of Health - *SUS* Hospital Information System (*SIH*/*SUS*)

### Number of deaths and lethality rate according to living region and age group

**Fig. 2:**
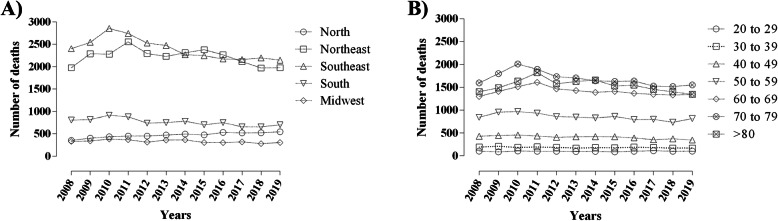
Distribution of deaths and mortality rate according to Brazilian regions and age group between 2008 and 2019. **a** living region; **b** age group. Source: Ministry of Health - *SUS* Hospital Information System (*SIH*/*SUS*)

A total of 74,180 deaths were observed between 2008 and 2019, representing 1.48% of the total deaths registered in Brazil in the same period. The lethality rate due to DM was 4.75. We also found 40,822 (55.03%) vs. 33,358 (44.97%) deaths for females and males when we compared sexes, respectively (*p* < 0.0001). The lethality rate was 6.5 vs. 5.8 in females and males, respectively. The highest death prevalence among Brazilian regions was found in the Southeast region (28,726; 38.72%) with a 5.33 lethality rate, followed by the Northeast region (26,636; 35.91%; 5.38 mortality rate). The distribution in all regions is shown in Fig. 2a.

Assessment of the number of deaths according to age groups showed that 27.27% (*n* = 20,230) occurred in those aged 70–79 years, followed by 24.92% (*n* = 18,485) in the age group > 80 years. Figure 2b shows the distribution in all age groups.

### Hospital length of stay and costs

Mean hospital length of stay due to DM was 6.2 ± 0.2 days. When we assessed hospital length of stay according to sex, males showed a mean of 6.5 ± 0.2 days and females 5.8 ± 0.2 days (*p* < 0.0001). In the Southeast region, 6.7 ± 0.1 days were recorded, followed by the North (6.3 ± 0.3 days), Northeast (6.1 ± 0.4 days), Midwest (5.6 ± 0.5 days) and South (5.3 ± 0.1 days); these were statistically significant (*p* < 0.0001), as shown in Table 1.

**Fig. 3:**
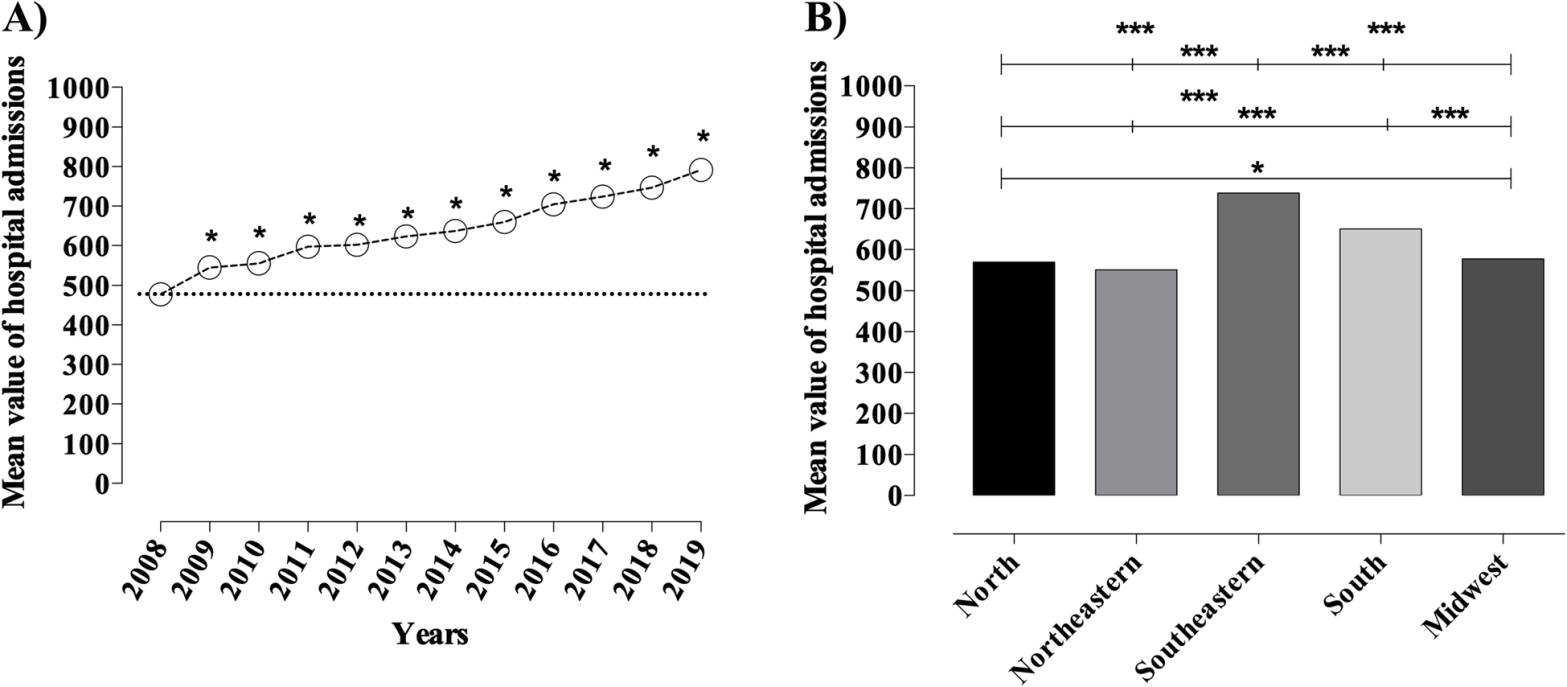
Hospital admission costs due to diabetes mellitus between 2008 and 2019. **a** Total costs among years. Statistical difference calculated by two-way ANOVA with a Tukey post hoc test; **b** Costs according to Brazilian regions. Values are expressed in mean. Statistical difference calculated by two-way ANOVA with Tukey post hoc test; * statistical difference between regions (* *p* = 0.02, Midwest vs. Northeast; ***p < 0.0001 North vs. Southeast, North vs. South, Southeast vs. Northeast, Southeast vs. South, Southeast vs. Midwest, Northeast vs. South and South vs. Midwest). Source: Ministry of Health - *SUS* Hospital Information System (*SIH*/*SUS*)

A total of R$993,256,834.60 were allocated for hospitalizations due to DM, with an average value of R$663.67 per hospitalization. A progressive increase in the mean value of hospital admissions over the years (R$313.49, 65.6%) was noted. This increased mean value of hospital admissions is in addition to the statistical difference in the mean value of hospitalization between regions (*p* < 0.0001 and *p* = 0.02), as shown in Fig. 3a and b, accordingly.

## Discussion

There was an increase in the number of hospitalizations and costs due to DM over the years, and majority of the cases (94.28%) were due to urgent causes. The Southeast region registered the highest incidence of hospitalizations (34.6%), as well as lethality rate. It was observed that females were more likely to be hospitalized in comparison to males. However, an inverse trend was noted over the years, and males showed a higher mortality rate than females. A progressive increase in hospitalization and lethality rate was also observed according to increased aging.

It is known that the number of people diagnosed with DM is increasing worldwide, and studies attribute this increase to urbanization and lifestyle changes [11, 12]. An increase in the number of hospitalizations due to DM was observed in this study. Telo et al. (2016) [13] noted an increase in DM prevalence in a systematic review. This is probably the first study to analyze epidemiological data on DM in Brazil in the last 12 years, especially regarding the number of hospitalizations and mortality rate according to age, gender and living region. In addition to the growing number of hospitalizations, we observed that more than 94% of cases were due to urgent causes, demonstrating that better care is still necessary for this population. Rosa et al. (2014) [14] analyzed the number of hospitalizations due to DM from 2008 and 2010 in Brazil, in which the authors observed that hospitalizations were associated to chronic DM general medical conditions in the majority of cases (89.7%). However, Rosa et al. analyzed the hospital admission for a short period of time, which differs from the current study.

When hospital admissions were grouped according to Brazilian regions, it was observed that the Southeast region presented the highest incidence of hospitalizations and lethality rate. This was followed by the Northeast, South, North and Midwest. According to the *Instituto Brasileiro de Geografia e Estatística* (IBGE) [15], this sequence corresponds to the population of these regions in the same order, which may justify our findings. A higher number of hospitalizations was noted in the Southeast region, perhaps due to its better access to healthcare, and consequently the greater number of diagnosed cases, and unlike the Northeast region where DM may be undiagnosed. Studies also estimate that 39% of adults with DM in South and Central Americas are undiagnosed [13].

Regarding sex, we observed a higher number of hospitalizations in women in comparison to men. Studies show that women with DM develop more serious complications, resulting in higher hospitalization numbers. In the last few decades women are being more careful with their health when compared to men. Females are more likely to seek medical help for weight loss therapy, decreasing one of the risk factors for hospitalizations [16].

This study observed a decrease in the number of hospitalizations due to DM in women, and increased in men. Moreover, males showed a higher mortality rate in comparison to females. Studies have shown that men are more likely to be diagnosed with DM compared to women, and this can be explained by hormonal predisposition. For instance, low testosterone levels in men are implicated in visceral fat deposition [17, 18], but this is poorly understood in literature [19]. The number of hospitalizations over the years generally showed a tendency to produce similar values, with no difference between sexes. This was also observed by Hilawe et al. (2013) [20] while analyzing differences by gender among individuals with DM in Africa.

A report published by the Surveillance of Risk and Protective Factors for Chronic Diseases Telephone Survey (*VIGITEL*) in 2013 [21] showed a higher prevalence (22.1%) of DM in the Brazilian age group ≥65 years, thus corroborating our study. This may be explained by the increased risk factors associated with aging and the global average life expectancy, such as the appearance of cardiovascular diseases (CVDs), physical inactivity and fat deposition [22]. According to Vitoi et al. (2015), the prevalence of DM in overweight older individuals was 55% higher in relation to eutrophic individuals in Brazil [23]. In a study performed by Schimidit et al. (2011) [24], the authors found a 2% increase on CVD mortality when CVDs are associated to DM.

Brazil spent an average of R$663.47 per hospitalization due to DM between 2008 and 2019. We also observed a progressive cost increase in all Brazilian regions, which may be explained by the increased hospitalizations over the years. In a study performed by Rosa et al. (2007) [25], they estimated an average cost per hospitalization around R$550 when the outcome was death, and R$287 when the subject was discharged between 1999 and 2011. Unfortunately, we did not analyze this separately; but it is possible to observe an increase in hospital expenses due to DM over the years.

This study was restricted only to the main diagnosis as presented in *SIH*/*SUS* by the ICD code, which is inadequate if little or no information about the several different conditions of the patients is available. It is important to highlight that the ICD diagnosis was confirmed after several visits to the hospitalized subject, which reduced changes of a wrong diagnosis. However, the ICD classification is the most used coding system by physicians, and it supports research and public health reports.

## Conclusions

The present study provides an update about patients who required hospitalization due to DM in Brazil. The study observed an increase in hospitalizations due to DM, as well as a progressive increase of hospital admissions and mortality rate according to aging which may lead to higher costs. There is a need for primary healthcare interventions to reduce the number of DM cases and hospitalizations due to complications in this group of patients in the country. Our report may help local and national healthcare managers to develop new strategies to prevent hospital admissions due to DM.

## Data Availability

The datasets analyzed during the current study are available in the Brazilian system of Hospital Information of the “*Sistema Único de Saúde*” repository, link: http://datasus.saude.gov.br/.
